# Giant Depolarizing Potentials Trigger Transient Changes in the Intracellular Cl^-^ Concentration in CA3 Pyramidal Neurons of the Immature Mouse Hippocampus

**DOI:** 10.3389/fncel.2018.00420

**Published:** 2018-11-20

**Authors:** Aniello Lombardi, Peter Jedlicka, Heiko J. Luhmann, Werner Kilb

**Affiliations:** ^1^Institute of Physiology, University Medical Center Mainz, Johannes Gutenberg University Mainz, Mainz, Germany; ^2^Interdisciplinary Centre for 3Rs in Animal Research, Faculty of Medicine, Justus Liebig University Giessen, Giessen, Germany; ^3^Institute of Clinical Neuroanatomy, Neuroscience Center, Goethe University Frankfurt, Frankfurt, Germany

**Keywords:** development, Cl^-^ homeostasis, gramicidin-perforated patch-clamp, post-synaptic currents, ionic plasticity, GABA(A) receptors

## Abstract

Giant depolarizing potentials (GDPs) represent a typical spontaneous activity pattern in the immature hippocampus. GDPs are mediated by GABAergic and glutamatergic synaptic inputs and their initiation requires an excitatory GABAergic action, which is typical for immature neurons due to their elevated intracellular Cl^-^ concentration ([Cl^-^]_i_). Because GABA_A_ receptors are ligand-gated Cl^-^ channels, activation of these receptors can potentially influence [Cl^-^]_i_. However, whether the GABAergic activity during GDPs influences [Cl^-^]_i_ is unclear. To address this question we performed whole-cell and gramicidin-perforated patch-clamp recordings from visually identified CA3 pyramidal neurons in immature hippocampal slices of mice at postnatal days 4–7. These experiments revealed that the [Cl^-^]_i_ of CA3 neurons displays a considerable heterogeneity, ranging from 13 to 70 mM (average 38.1 ± 3.2 mM, *n* = 36). In accordance with this diverse [Cl^-^]_i_, GDPs induced either Cl^-^-effluxes or Cl^-^-influxes. In high [Cl^-^]_i_ neurons with a negative Cl^-^-driving force (DF_Cl_) the [Cl^-^]_i_ decreased after a GDP by 12.4 ± 3.4 mM (*n* = 10), while in low [Cl^-^]_i_ neurons with a positive DF_Cl_ [Cl^-^]_i_ increased by 4.4 ± 0.9 mM (*n* = 6). Inhibition of GDP activity by application of the AMPA receptor antagonist CNQX led to a [Cl^-^]_i_ decrease to 24.7 ± 2.9 mM (*n* = 8). We conclude from these results, that Cl^-^-fluxes via GABA_A_ receptors during GDPs induced substantial [Cl^-^]_i_ changes and that this activity-dependent ionic plasticity in neuronal [Cl^-^]_i_ contributes to the functional consequences of GABAergic responses, emphasizing the concept that [Cl^-^]_i_ is a state- and compartment-dependent parameter of individual cells.

## Introduction

Spontaneous neuronal activity transients are a hallmark of developing neuronal systems and play an essential role for several developmental processes like neuronal migration, myelinization, cortical regionalization, or the establishment of neuronal connectivity (for review Spitzer, 2006; Ben-Ari and Spitzer, 2010; Griguoli and Cherubini, 2017; Kirischuk et al., 2017). Such spontaneous activity transients can be generated in the sensory periphery, but are also intrinsic to developing neuronal networks (for review Ben Ari, 2001; Khazipov and Luhmann, 2006; Blankenship and Feller, 2010; Kilb et al., 2011). The fact that even synaptic networks of cultured neurons develop correlated activity transients (Rolston et al., 2007; Sun et al., 2010) suggests that the formation of circuits capable of mediating recurrent activity is probably an innate feature of the neuronal nature. One striking example for spontaneous, repetitive activity transients are hippocampal giant depolarizing potentials (GDPs) (Ben-Ari et al., 1989). Such GPDs have also been observed in the immature neocortex (Allene et al., 2008), thalamus (Pangratz-Fuehrer et al., 2007), and the embryonic spinal cord (Czarnecki et al., 2014).

Giant depolarizing potentials represent recurrent, synaptically evoked suprathreshold depolarizations, which rely on GABAergic and glutamatergic transmission (Ben-Ari et al., 1989; Blankenship and Feller, 2010). GDPs require a depolarizing GABAergic action (Sipila et al., 2006b; Cherubini et al., 2011). Such depolarizing GABAergic responses are typical for immature neurons (for review Ben-Ari, 2002; Ben-Ari et al., 2012; Watanabe and Fukuda, 2015; Kirmse et al., 2018) and reflect an elevated intracellular Cl^-^ concentration ([Cl^-^]_i_) maintained by a Cl^-^ uptake via the isoform 1 of the Na^+^-dependent K^+^-Cl^-^-Cotransporter (NKCC1) (Rohrbough and Spitzer, 1996; Yamada et al., 2004; Achilles et al., 2007; Blaesse et al., 2009). The membrane depolarization via synaptic and extrasynaptic GABA_A_ receptors, which is augmented via an activation of persistent Na^+^ currents (Sipila et al., 2006a; Valeeva et al., 2010), facilitates the onset of neuronal activity underlying GDPs (Sipila et al., 2005). Subsequently, hippocampal neurons start burst firing and this activity is transmitted via GABA_A_ and AMPA receptors to adjacent interneurons and pyramidal neurons (Khazipov et al., 1997; Bolea et al., 1999). A subset of GABAergic interneurons, with a high synaptic connectivity and a putative origin in the medial ganglionic eminence, serve as hub neurons with specific importance during the generation of GDPs (Bonifazi et al., 2009; Wester and McBain, 2016).

In accordance with these observations, GDPs are characterized by an initial phase of depolarizing GABAergic post-synaptic currents (PSCs) and subsequent AMPA mediated PSCs which synergistically drive the depolarization (Khalilov et al., 2015). When the depolarization surpasses the GABA equilibrium potential (E_GABA_) the GABAergic currents become outwardly directed, promoting an inhibitory action of GABA that limits the amount of depolarization/activation and thus the synchronization between pyramidal neurons (Khalilov et al., 2015). Finally, activation of Ca^2+^-dependent K^+^ channels and post-synaptic GABA_B_ receptors decreases the excitability and the firing frequency of pyramidal neurons (Sipila et al., 2006a; Khalilov et al., 2017), which reduce AMPA and GABA post-synaptic potentials (PSPs) and thus terminates GDPs.

Since GABA_A_ receptors are ligand-gated anion-channels with a high permeability for Cl^-^ ions (Farrant and Kaila, 2007), Cl^-^-fluxes through activated GABA_A_ receptors can influence [Cl^-^]_i_ on a shorter time scale (Kaila et al., 1989; Bracci et al., 2001; Isomura et al., 2003; Jedlicka and Backus, 2006; Jedlicka et al., 2011; Lillis et al., 2012; Sato et al., 2017). The resulting activity-dependent [Cl^-^]_i_ increase upon massive GABAergic stimulation, in combination with the HCO_3_^-^ permeability of GABA_A_ receptors, leads to a shift from hyperpolarizing/inhibitory to depolarizing/excitatory GABAergic action (Staley et al., 1995; Sun et al., 2001; Isomura et al., 2003, but see Kaila et al., 1997 for more complex events involved in this hyperpolarizing-depolarizing shift). This process has been termed ionic plasticity (Blaesse et al., 2009; Raimondo et al., 2012; Kaila et al., 2014a). The relation between Cl^-^ influx, dendritic volume/morphology and the capacity of Cl^-^ extrusion systems determines the size of such activity-dependent Cl^-^ transients (Staley and Proctor, 1999; Wright et al., 2011; Mohapatra et al., 2016). In the immature system, with high [Cl^-^]_i_ and depolarizing GABAergic events, the GABA-mediated Cl^-^ efflux causes a transient decline in [Cl^-^]_i_ that temporarily attenuates the amplitude of subsequent GABAergic responses, thereby reducing or omitting possible excitatory effects (Achilles et al., 2007; Gonzalez-Islas et al., 2010; Kolbaev et al., 2011b). However, it has remained unclear whether GDPs lead to detectable [Cl^-^]_i_ changes.

In order to elucidate whether the massive GABAergic activity during a GDP influences [Cl^-^]_i_ in the immature hippocampus, we performed whole-cell and gramicidin-perforated patch-clamp recordings from visually identified CA3 pyramidal neurons in hippocampal slices from mice at postnatal day 4–7. These experiments revealed that the [Cl^-^]_i_ in pyramidal neurons is altered following a GDP. The direction and amount of [Cl^-^]_i_ changes depends on the cell’s individual [Cl^-^]_i_, with a [Cl^-^]_i_ decrease in high [Cl^-^]_i_ neurons and a [Cl^-^]_i_ increase at low [Cl^-^]_i_ neurons. Inhibition of GDP activity leads to a decreased [Cl^-^]_i_. These results indicate that ongoing GDP activity induces ionic plasticity in immature hippocampal neurons and emphasize the concept that neuronal [Cl^-^]_i_ has to be considered as a state- and compartment-dependent parameter of individual cells (Wright et al., 2011).

## Materials and Methods

### Slice Preparation

All experiments were conducted in accordance with EU directive 86/609/EEC for the use of animals in research and the NIH Guide for the Care and Use of Laboratory Animals, and were approved by the local ethical committee (Landesuntersuchungsanstalt RLP, Koblenz, Germany). All efforts were made to minimize the number of animals and their suffering. Time pregnant C57Bl/6 mice were obtained from Janvier Labs (Saint Berthevin, France) and housed in the local animal facility. Newborn pups of postnatal days [P] 4–7 were deeply anesthetized with enflurane (Ethrane, Abbot Laboratories, Wiesbaden, Germany). After decapitation, the brains were quickly removed and immersed for 2–3 min in ice-cold standard artificial cerebrospinal fluid (ACSF, composition see below). Horizontal slices (400 μm thickness) including the hippocampus were cut on a vibratome (Microm HM 650 V, Thermo Fischer Scientific, Schwerte, Germany). The slices were stored in an incubation chamber filled with oxygenated ACSF at room temperature before they were transferred to the recording chamber.

### Solutions and Drugs

The bathing solution consisted of (in mM) 125 NaCl, 25 NaHCO_3_, 1.25 NaH_2_PO_5_, 1 MgCl_2_, 2 CaCl_2_, 2.5 KCl, 10 glucose and was equilibrated with 95% O_2_/5% CO_2_ at least 1 h before use (pH 7.4, osmolarity 306 mOsm). Two pipette solutions for whole-cell recordings were used: for a pipette Cl^-^ concentration ([Cl^-^]_p_) of 10 mM the solution was composed of (in mM) 128 K-gluconate, 2 KCl, 4 NaCl, 1 CaCl_2_, 11 EGTA, 10 K-HEPES, 2 Mg^2^-ATP, 0.5 Na-GTP, and 2 lidocaine-*N*-ethyl chloride (pH adjusted to 7.4 with KOH and osmolarity to 306 mOsm with sucrose). For a [Cl^-^]_p_ of 50 mM the solution contained (in mM) 86 K-gluconate, 44 KCl, 4 NaCl, 1 CaCl_2_, 11 EGTA, 10 K-HEPES, 2 Mg2-ATP, 0.5 Na-GTP. Pipette solution for gramicidin-perforated patch experiments consisted of (in mM) 10 Na-gluconate, 120 KCl, 1 CaCl_2_, 2 MgCl_2_, 11 EGTA, and 10 K-HEPES. For gramicidin-perforated patch-clamp recordings 10 μg/ml Gramicidin D (Sigma, St. Louis, MO, United States) was added from a stock solution (2 mg/ml in DMSO) on the day of experiment. Gramicidin D, bumetanide, and lidocaine-*N*-ethyl chloride was obtained from Sigma-Aldrich and 6-Cyano-7-nitroquinoxaline-2,3-dione (CNQX) was obtained from Biotrend (Cologne, Germany). CNQX and bumetanide were used from a dimethylsulfoxide (DMSO, Sigma-Aldrich) stock solution. The DMSO concentration of the final solution never exceeded 0.1%.

### Data Acquisition and Analysis

Whole-cell and gramicidin-perforated patch-clamp recordings were performed as described previously (Kyrozis and Reichling, 1995; Kolbaev et al., 2011b) at 31 ± 1°C in a submerged-type recording chamber attached to the fixed stage of a microscope (BX51 WI, Olympus). Pyramidal neurons in the stratum pyramidale of the CA3 region were identified by their location and morphological appearance in infrared differential interference contrast image. Patch-pipettes (5–12 MΩ) were pulled from borosilicate glass capillaries (2.0 mm outside, 1.16 mm inside diameter, Science Products, Hofheim, Germany) on a vertical puller (PP-830, Narishige) and filled with the pipette solutions (composition see above).

Signals were recorded with a discontinuous voltage-clamp/current-clamp amplifier (SEC05L, NPI, Tamm, Germany), low-pass filtered at 3 kHz and stored and analyzed using an ITC-1600 AD/DA board (HEKA) and TIDA software. All voltages were corrected *post hoc* for liquid junction potentials of -9 mV for 10 mM [Cl^-^]_p_, -6 mV for 50 mM [Cl^-^]_p_, and -3 mV for the perforated-patch solution (Achilles et al., 2007). Input resistance and capacitance were determined from a series of hyperpolarizing current steps. Action potential amplitude was calculated from the threshold (as determined by eye) and action potential duration was measured at half-maximal amplitude. Spontaneous post-synaptic currents (sPSCs) were detected and analyzed according to their amplitude and shape by appropriate settings using Minianalysis Software (Synaptosoft, Fort Lee, NJ, United States). Charge transfer is the total amount of charges that flow during an event. For PSCs it was determined in Minianalysis by integration of the currents between calculated onset and termination time points. Charge transfer of GDPs was determined in TIDA by integration of the current deflection from the holding current between the starting and endpoint of a GDP, as defined by eye.

The GABA reversal potential (E_GABA_) was determined from the GABAergic currents induced by focal pressure application of 100 μM GABA via a micropipette (tip diameter ca. 1–2 μm, placed 50–100 μm from the soma in the stratum radiatum, puff duration 5–10 ms) during a voltage ramp protocol (from -3 to -63 mV; Figures 3A,B). For this purpose the current of a control voltage ramp was subtracted from the current of the voltage ramp delivered in the presence of GABA. The voltage ramp was applied during a quasi-stationary phase of the GABAergic response (Figure 3B). The E_m_ value at which this differential current reverses was considered as E_GABA_ (Figures 3C–E). Because for the central aim of this study it was not possible to pharmacologically block voltage-dependent Na^+^ currents with TTX, the voltage ramp protocol was preceded by a 100 ms long depolarizing phase at -3 mV to inactivate voltage-dependent Na^+^ currents (Figure 3B).

In order to take the contribution of HCO_3_^-^ ions to the reversal potential of GABA_A_ receptors into account (Farrant and Kaila, 2007), we calculated [Cl^-^]_i_ from E_GABA_ with the Goldman-Hodgkin-Katz equation:

$$E_{\mathrm{GABA}}=\frac{\mathrm{RT}}{\mathrm{ZF}}*\ln\left(\frac{P_{\mathrm{Cl}}{\left[Cl^{-}\right]}_{e}+P_{\mathrm{HCO3}}{\left[HCO_{3}^{-}\right]}_{e}}{P_{\mathrm{Cl}}{\left[Cl^{-}\right]}_{i}+P_{\mathrm{HCO3}}{\left[HCO_{3}^{-}\right]}_{i}}\right)$$

For the calculation of [Cl^-^]_i_ from E_GABA_ we used a [Cl^-^]_e_ of 133.5 mM, an extracellular HCO_3_^-^ concentration ([HCO_3_^-^]_e_) of 24 mM and a [HCO_3_^-^]_i_ of 14.1 mM. The [HCO_3_^-^]_i_ was calculated with the Henderson–Hasselbalch equation using a CO_2_ pressure of 38 mmHg (corresponding to 5% CO_2_ at 760 Torr), an intracellular pH of 7.2 (Ruusuvuori et al., 2010), a Henry coefficient of 0.318 and a pKs of 6.128 (Mitchell et al., 1965). A relative HCO_3_^-^ permeability (P_HCO_) of 0.44, which has been determined for GABA_A_ receptors in hippocampal neurons (Fatima-Shad and Barry, 1993), was used and P_Cl_ was defined as 1. The driving-force of Cl^-^ (DF_Cl_) was calculated from the difference between the average E_m_ during a GDP and E_Cl_ (DF_Cl_ = E_m_ – E_GABA_). To calculate GABAergic (g_GABA_) and glutamatergic conductances (g_Glu_) the peak amplitudes of GDP associated currents (I_GDP_) were divided by the estimated driving force at the given holding potentials using Ohm’s law [for GABA: g_GABA_ = I_GDP_/(E_m_ - E_Rev^GABA^_); for glutamate: g_Glu_ = I_GDP_/(E_m_ - E_Rev^GABA^_)].

All values are given as mean ± SEM. If not explicitly noted, Student’s *t*-test was used for statistical analysis (Systat 11). Significance was assigned at *p*-levels of 0.05 (^∗^), 0.01 (^∗∗^), and 0.001 (^∗∗∗^).

### Microcontroller-Based GDP Detection

To enable the online determination of [Cl^-^]_i_ at distinct latencies after a GDP we used a microcontroller (Arduino Uno^1^) connected to the inputs and outputs of the NPI amplifier. A threshold crossing algorithm comparing a floating average of 50 E_m_ datapoints (to avoid triggering by single action potential) with a manually preset threshold potential was used to detect a GDP (Figure 3F). The time point when E_m_ falls below the threshold was defined as end of a GDP. After a defined interval (0.1–20 s) the microcontroller provided a signal that switches the amplifier to voltage-clamp mode and delivers the ramp protocol used for the determination of E_GABA_ (Figures 3A–E). The used program code for the Arduino microcontroller is available at https://forum.arduino.cc/index.php?topic=564489.0.

### Compartmental Modeling

For morphological reconstruction some CA3 pyramidal cells were filled with biocytin (0.5–1%, Sigma-Aldrich) under whole-cell conditions as described in detail before (Horikawa and Armstrong, 1988; Schröder and Luhmann, 1997). Reconstruction and morphological analysis of the biocytin-labeled neurons were performed from 60x oil-immersion images using Fiji ^2^. A reconstructed CA3 pyramidal cell was imported into the NEURON simulation program ^3^ (Figures 5A,B). The following passive parameters were used: *R_a_* (specific axial resistance) = 34.5 Ωcm; *R_m_* (specific membrane resistance) = 2 kΩcm^2^; *C_m_* (specific membrane capacitance) = 1 μFcm^-2^.

GABA_A_ synapses were simulated as a post-synaptic parallel Cl^-^ and HCO_3_^-^ conductance with exponential rise and exponential decay (Jedlicka et al., 2011):

$$I_{\mathrm{GABA}}=I_{\mathrm{Cl}}+I_{\mathrm{HCO3}}=1/(1+\mathrm{P)}\cdot g_{\mathrm{GABA}}\cdot (V-E_{\mathrm{Cl}})+P/(1+P)\cdot g_{\mathrm{GABA}}\cdot (V-E_{\mathrm{HCO}3})$$

where P is a fractional ionic conductance that was used to split the GABA_A_ conductance (g_GABA_) into Cl^-^ and HCO_3_^-^ conductance. E_Cl_ and E_HCO3_ were calculated from Nernst equation. The GABA_A_ conductance was modeled using a two-term exponential function, using separate values of rise time (0.5 ms) and decay time (80 ms) (Santhakumar et al., 2005). Parameters used in our simulations were as follows: [Cl^-^]_o_ = 133.5 mM, [HCO3^-^]_i_ = 14.1 mM, [HCO3^-^]_o_ = 24 mM, temperature = 31°C, *p* = 0.44 (Fatima-Shad and Barry, 1993).

For the experiments used to model the impact of access resistance (R_s_) or dendritic filtering on the determination of the reversal potential we considered static [Cl^-^]_i_ and [HCO_3_^-^]_i_ ([Cl^-^]_i_ = 30 mM; [HCO_3_^-^]_i_ = 14.4 mM) and implemented a single-electrode voltage-clamp process to the soma, using R_s_ values of 0.5, 5, 10, 20, and 40 MΩ. A GABA synapse with a decay time of 100 ms and a peak conductance of 10 nS was used to emulate the GABA application protocol.

For the modeling of the GDP-induced [Cl^-^]_i_ and [HCO_3_^-^]_i_ changes we calculated ion diffusion and uptake by standard compartmental diffusion modeling (De Schutter and Smolen, 1998; De Schutter, 2010; Mohapatra et al., 2014, 2016). To simulate intracellular Cl^-^ and HCO_3_^-^ dynamics, we adapted our previously published model (Jedlicka et al., 2011). Longitudinal Cl^-^ and HCO_3_^-^ diffusion along dendrites was modeled as the exchange of anions between adjacent compartments. For radial diffusion, the volume was discretized into a series of four concentric shells around a cylindrical core (De Schutter and Smolen, 1998) and Cl^-^ or HCO_3_^-^ was allowed to flow between adjacent shells (Hines and Carnevale, 2000). The free diffusion coefficient of Cl^-^ inside neurons was set to 2 μm^2^/ms (Kuner and Augustine, 2000). Since the cytoplasmatic diffusion constant for HCO_3_^-^ is to our knowledge unknown, we also used a value of 2 μm^2^/ms. To simulate transmembrane transport of Cl^-^ and HCO_3_^-^, we implemented an exponential relaxation process for [Cl^-^]_i_ and [HCO_3_^-^]_i_ to resting levels [Cl^-^]_i_^rest^ or [HCO_3_^-^]_i_^rest^ with a time constant τ_Ion_.

$$\frac{d{\left[{\mathrm{Ion}}^{-}\right]}_{i}}{\mathrm{dt}}=\frac{{\left[{\mathrm{Ion}}^{-}\right]}_{i}^{\mathrm{rest}}-e{\left[{\mathrm{Ion}}^{-}\right]}_{i}}{\tau_{\mathrm{Ion}}}$$

Cl^-^ transport was modeled as bimodal process, for [Cl^-^]_i_ < [Cl^-^]_i_^rest^ τ was set to 174s to emulate an NKCC1-like Cl^-^ transport mechanism. For [Cl^-^]_i_ > [Cl^-^]_i_^rest^ τ was set to 321s to emulate passive Cl^-^ efflux (both values obtained from unpublished experiments on immature rat CA3 hippocampal neurons). The impact of GABAergic Cl^-^ currents on [Cl^-^]_i_ and [HCO_3_^-^]_i_ was calculated as:

$$\frac{d{\left[{\mathrm{Ion}}^{-}\right]}_{i}}{\mathrm{dt}}=\frac{1}{F}\frac{I_{\mathrm{Ion}}}{\mathrm{volume}}$$

To simulate the GABAergic activity during a GDP, 534 GABA synapses with a peak conductance of 0.789 nS and a decay of 80 ms were randomly distributed in the dendritic compartment of the reconstructed neurons. This number of GABAergic inputs generates a charge transfer of 88 pA (at initial [Cl^-^]_i_ of 10 mM and under VC conditions at 0 mV) similar to the charge transfer recorded experimentally under this condition (see section “Properties of GDPs”). To simulate AMPA synapses, additional 107 Exp2syn processes with a peak conductance of 0.509 nS, a reversal potential of 0 mV, a rise time of 0.1 ms, and a decay of 11 ms were randomly distributed. GABA and AMPA inputs were activated stochastically using a normal distribution that emulates the distribution of glutamatergic/GABAergic PSCs observed in the present study. We analyzed the mean [Cl^-^]_i_ and [HCO_3_^-^]_i_ of all dendrites to simulate the experimental procedure for E_GABA_ determination, in which GABA was applied to the dendritic compartment.

## Results

### Properties of the Recorded Cells

The average resting membrane potential (RMP) of the CA3 pyramidal cells under whole cell condition was -52.1 ± 0.8 mV (*n* = 116), their input resistance (R_in_) at RMP was 0.9 ± 0.1 GΩ and their membrane time constant amounted to 86 ± 8.3 ms, corresponding to a membrane capacitance of 152 ± 24.3 pF. Upon depolarization above a membrane potential (E_m_) of -42.4 ± 0.68 mV, these cells were capable to fire action potentials with an amplitude of 54.9 ± 1.3 mV (*n* = 72) and a duration of 2.7 ± 0.4 ms. Comparable results were observed under gramicidin-perforated patch conditions. In these experiments the average resting membrane potential was -53.6 ± 0.24 mV (*n* = 24), the input resistance was 1.75 ± 0.13 GΩ and the membrane time constant amounted to 105.9 ± 11.4 ms, corresponding to a membrane capacitance of 65 ± 9.1 pF. Upon E_m_ depolarization above -46.2 ± 1.2 mV these cells fired action potentials with an amplitude of 36 ± 2 mV (*n* = 22) and a duration of 3.6 ± 0.4 ms.

### Properties of GDPs

**FIGURE 1:**
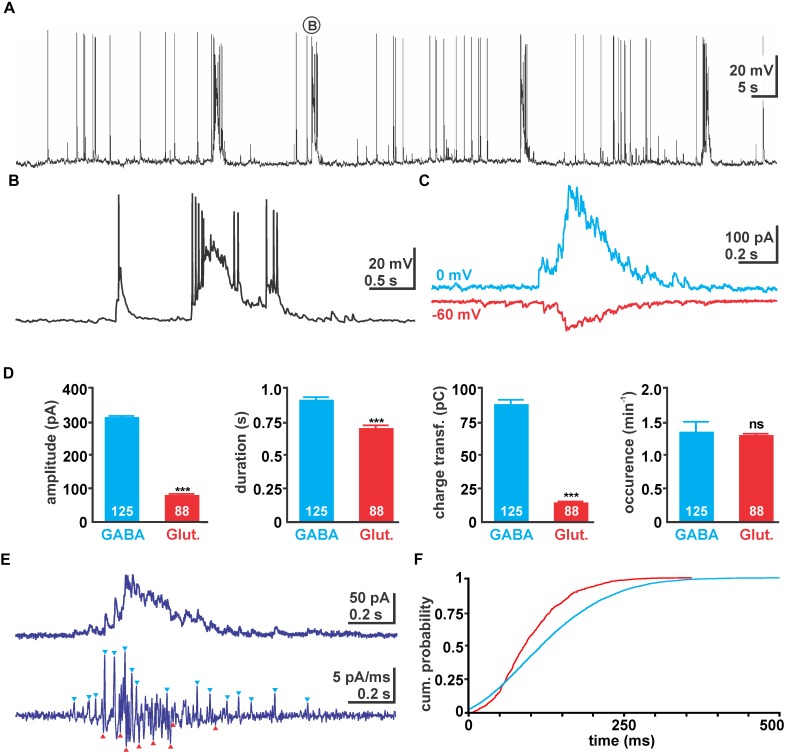
Properties of giant depolarizing potential (GDP) activity in immature hippocampal slices. **(A)** Typical voltage traces recorded with 50 mM [Cl^-^]_*p*_. Note the occurrence of massive depolarizing shifts triggering several action potentials (APs). **(B)** One typical GDP as indicated in **(A)** in higher temporal resolution. **(C)** Typical GDP associated currents recorded with 10 mM [Cl^-^]_*p*_ at 0 mV (blue traces) and –60 mV (red traces), reflecting GABAergic and glutamatergic currents, respectively. Note that the traces were recorded from the same neuron in two independent experiments and have been manually aligned. **(D)** Statistical evaluation of absolute current amplitude, duration, charge transfer, and occurrence of GDP associated GABAergic and glutamatergic currents. Glutamatergic currents have smaller amplitude and mediate less charge transfer. Values in the bar diagrams represent mean ± SEM, number of experiments are given in the bars, ^∗∗∗^ indicates a *p*-level of <0.001. **(E)** Typical current recording using 10 mM [Cl^-^]_*p*_ at –30 mV. Since from this trace GABAergic outward currents and glutamatergic inward currents could not be separated easily (upper trace), the first derivative of this recording was calculated (lower trace). From this trace several outward and inward currents could be identified (blue/red arrowheads). **(F)** Cumulative probability plot of the identified GABAergic (blue, 4004 events) and glutamatergic (red, 699 events) post-synaptic events (PSCs) during a GDP calculated from 105 GDPs in 15 recordings. Note the earlier onset of GABAergic PSCs and that glutamatergic PSCs terminated much earlier.

Giant depolarizing potentials were present in 96 of in total 111 cells (corresponding to 86.5%) from 73 slices/44 animals investigated with different recording conditions. In the first set of experiments we used a high Cl^-^ pipette solution ([Cl^-^]_p_ = 50 mM) to characterize the properties of spontaneous network events. Under this condition in all 13 cells (from 13 slices/6 animals) spontaneous massive depolarizing events with an average amplitude of 24.9 ± 1.2 mV (*n* = 130 GDPs in 13 cells) and a duration of 1.24 ± 0.04 s were observed (Figures 1A,B). While these depolarizing events were subthreshold in three cells, they reliably evoked 6.4 ± 0.4 APs (*n* = 104 GDPs in 10 cells) in the remaining cells. Since the amplitude, duration and appearance of these depolarizing events mimic the properties of GDPs (Ben-Ari et al., 1989; Khalilov et al., 2015), we considered them as GDPs. These GDPs occurred at a rate of 1.3 ± 0.13 min^-1^ (*n* = 13 cells).

To investigate the contribution of synaptic events underlying these GDPs, we next performed voltage-clamp recordings using a [Cl^-^]_p_ of 10 mM. For this purpose GABAergic and glutamatergic synaptic events were isolated at 0 mV (close to the reversal potential of glutamatergic currents) and at -60 mV (close to the calculated reversal potential of GABAergic currents at a [Cl^-^]_p_ of 10 mM), respectively. At a holding potential of 0 mV in total 125 GDPs associated inward currents (I_GDP_) were identified (*n* = 17 cells), occurring at a rate of 1.35 ± 0.15 min^-1^ (Figure 1C). These I_GDP_ had an average amplitude of 309.5 ± 3.5 pA (*n* = 125), a duration of 0.91 ± 0.02 s and conveyed an average charge transfer of 88 ± 3.3 pC (Figure 1D). At a holding potential of -60 mV in total 88 I_GDP_ from 15 cells could be recorded. They had an average amplitude of 79.3 ± 3.7 pA (*n* = 88), a duration of 0.7 ± 0.02 s, conveyed an average charge transfer of 14.6 ± 0.7 pC, and occurred at a rate of 1.3 ± 0.2 min^-1^ (Figures 1C,D). These peak currents correspond to conductances of 5.1 ± 0.1 nS for the GABAergic and 1.3 ± 0.06 nS for the glutamatergic GDP component.

In order to analyze the temporal relation between GABAergic and glutamatergic synaptic inputs during a GDP, we performed voltage-clamp recordings at -30 mV, which in theory would enable identification of glutamatergic inward and GABAergic outward currents. However, as from the raw current traces GABAergic outward currents and glutamatergic inward current could not be separated (Figure 1E), we had to identify the onset of GABAergic and glutamatergic events from the first derivative of the current trace (Figure 1E). The cumulative probability distribution of those identified GABAergic (*n* = 4003 events) and glutamatergic events (*n* = 699) revealed that glutamatergic events start slightly delayed to GABAergic synaptic inputs and also terminated significantly earlier (*p* < 0.001, Kolmogorov–Smirnov test, 2351 events) (Figure 1F), although these events reflect only a fraction of all synaptic events during a GDP.

### Relation Between GDPs and Spontaneous Synaptic Events

**FIGURE 2:**
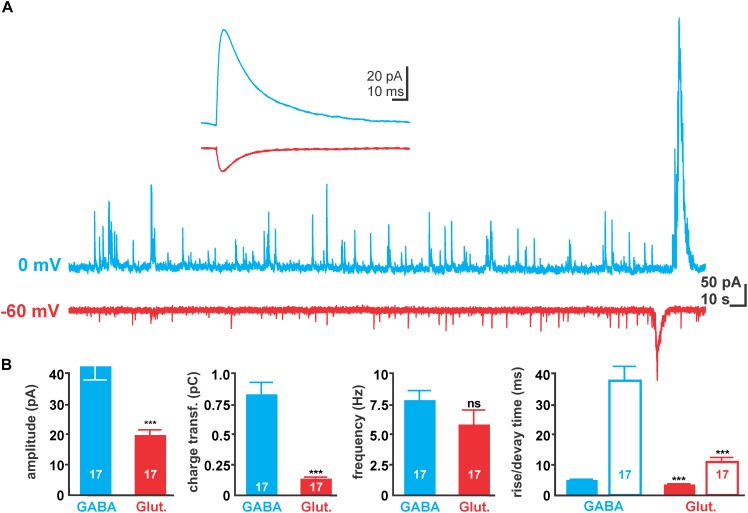
Properties of spontaneous synaptic activity in immature hippocampal slices. **(A)** Typical spontaneous post-synaptic currents (sPSCs) recorded with 10 mM [Cl^-^]_p_ at 0 mV (blue traces) and –60 mV (red traces), reflecting GABAergic and glutamatergic sPSCs, respectively. Note the large current deflections at the end of the traces, which represent GDP-associated currents and were excluded from analysis. **(B)** Statistical evaluation of amplitude, charge transfer, frequency, and kinetic properties of GABAergic and glutamatergic sPSCs. Glutamatergic currents have smaller amplitude and mediate less charge transfer due to their smaller amplitude and faster decay time (open bars). Bars represent mean ± SEM, numbers of experiments are given in the bars, ^∗∗∗^ indicates a *p*-level of <0.001.

In order to estimate how much synaptic inputs underlie a GDP, we next analyzed the properties of spontaneous post-synaptic events (sPSCs) that occurred clearly outside of GDPs (Figure 2A). Voltage-clamp recordings using a [Cl^-^]_p_ of 10 mM revealed that at -60 mV (close to the calculated reversal potential of GABAergic currents) sPSCs had an average amplitude of 18.3 ± 1.3 pA (*n* = 17 cells), a rise time of 3.5 ± 0.2 ms, a decay time of 10.8 ± 1.5 ms and a charge transfer of 131.5 ± 14.4 fC. These putatively glutamatergic sPSCs occurred at a frequency of 5.8 ± 1.2 Hz (Figure 2B).

At a holding potential of 0 mV (close to the calculated reversal potential of glutamatergic currents) putatively GABAergic sPSCs (Figure 2A) with a significantly (*p* < 0.001) larger amplitude of 46.9 ± 3.1 pA (*n* = 17 cells), longer rise (4.9 ± 0.2 ms) and decay (37.3 ± 3.7 ms) times and a larger charge transfer (872.3 ± 96.2 fC) were observed (Figure 2B). These putatively GABAergic sPSCs occurred at a frequency of 7.8 ± 0.8 Hz.

The average amplitudes of GABAergic and glutamatergic sPSCs correspond under these conditions to a unitary conductance of 0.78 and 0.3 nS, respectively. Comparison of charge transfer at holding potentials of 0 mV suggests that 101 GABAergic synaptic inputs mediate the same charge transfer as the GABAergic component of an average GDP. And the comparison of charge transfer at holding potentials of -60 mV suggests that 107 glutamatergic synaptic inputs mediate the same charge transfer as the glutamatergic component of an average GDP.

### GDPs Induced Transient [Cl^-^]_i_ Alterations

As a variety of studies demonstrated that excessive GABAergic stimulation can alter [Cl^-^]_i_ (Kaila et al., 1989; Staley et al., 1995; Jedlicka and Backus, 2006; Achilles et al., 2007; Kolbaev et al., 2011b), we assumed that the Cl^-^-fluxes underlying the GABAergic currents during a GDP may be sufficient to induce significant alterations in [Cl^-^]_i_. To address this question, we determined the [Cl^-^]_i_ shifts after a GDP had occurred. In order to maintain the [Cl^-^]_i_ undisturbed the experiments were performed under gramicidin-perforated patch-clamp conditions (Kyrozis and Reichling, 1995). These experiments demonstrated that the average [Cl^-^]_i_ of CA3 pyramidal neurons amounted to 38.1 ± 3.2 mM (*n* = 36), however with a considerable scatter between ca. 13 and 70 mM. Application of the NKCC1 inhibitor bumetanide (10 μM) significantly (*p* < 0.001) reduced the [Cl^-^]_i_ to 21.4 ± 2.7 mM (*n* = 9). In the presence of bumetanide a slow [Cl^-^]_i_ decline with a time constant of 280s occurred, indicating that passive Cl^-^-fluxes are rather small. In addition, the coefficient of variation (CV) in [Cl^-^]_i_ droped from 0.51 under control conditions to 0.37 in the presence of bumetanide, indicating a more homogenous distribution of [Cl^-^]_i_.

**FIGURE 3:**
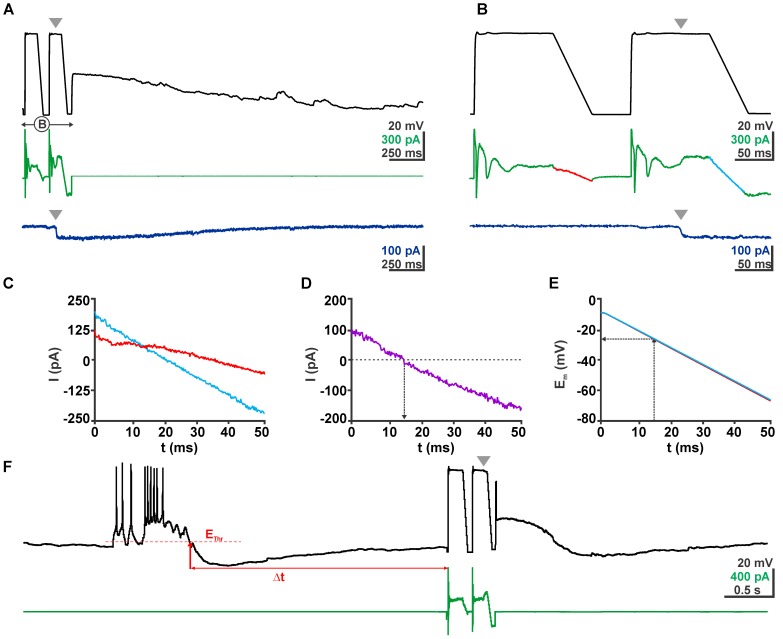
Determination of E_GABA_ using a voltage ramp protocol. **(A)** Voltage (black trace) and current (green trace) traces of a typical clamp protocol used to determine E_GABA_. Voltage ramps between –3 and –63 mV were given from a 100 ms long depolarizing phase to inactivate fast Na^+^ currents. The second voltage ramp was delivered during the quasi-stationary phase of GABAergic response evoked by a short application of 100 μM GABA pulse (gray arrowhead). The dark blue trace in the bottom depicts the current response of the same cell without a voltage ramp protocol to illustrate the stationarity of the GABAergic current during the voltage ramp. **(B)** The ramp interval as shown in **(A)** at a higher temporal resolution. **(C)** Current traces from **(B)** recorded in the absence (red) and presence (blue) of GABA. **(D)** The difference between both current traces reverses at 13 ms. **(E)** Voltage traces recorded in the absence (red) and presence (blue) of GABA. Note that both traces are virtually identical. From these traces the reversal potential was determined. **(F)** Voltage and current traces of an experiment illustrating the procedures of a GDP triggered ramp protocol. From a continuous recording in current-clamp mode the microcontroller identified a GDP that crossed the threshold (E_Thr_), the end of this GDP was determined as the time point when E_m_ recovers to E_Thr_. After a defined latency (Δt) the microcontroller provided a pulse to the amplifier to switch to VC mode and run the ramp protocol.

Under gramicidin-perforated patch-clamp conditions GDPs had an average depolarization of -46.9 ± 0.5 mV (*n* = 119 GDPs from 19 neurons) and lasted for 1.09 ± 0.07 s. To quantify possible GDP-induced [Cl^-^]_i_ changes, [Cl^-^]_i_ was determined between 0.5 and 10 s after the termination of a GDP by a ramp-protocol under voltage-clamp conditions (Figure 3). Beginning and end of each GDP were detected by threshold-crossing algorithms implemented in a microcontroller (Figure 3F).

**FIGURE 4:**
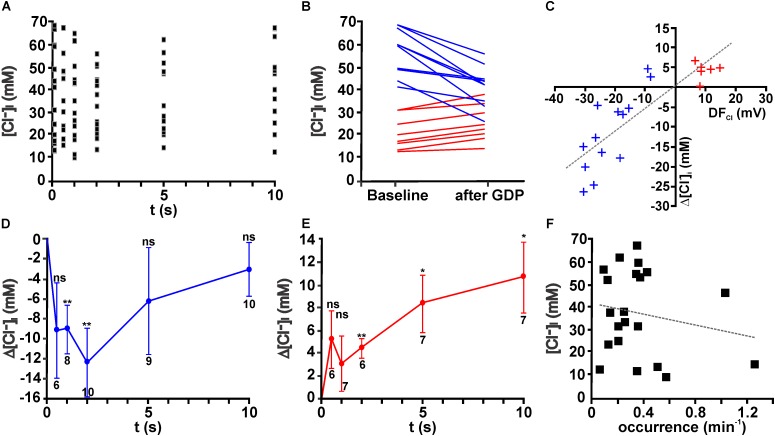
Giant depolarizing potentials lead to substantial [Cl^-^]_i_ changes. **(A)** Scatter plot of [Cl^-^]_i_ determined at given intervals after a GDP in 19 neurons. Note that the [Cl^-^]_i_ values tend to decrease between 1 and 5 s. **(B)** [Cl^-^]_i_ of individual neurons at baseline conditions and 1–2 s after a GDP. Note the obvious alteration in [Cl^-^]_i_. Cells that respond with a [Cl^-^]_i_ increase are represented by red lines and [Cl^-^]_i_ decreases by blue lines. **(C)** Plot of the GDP induced [Cl^-^]_i_ change (Δ[Cl^-^]_i_) against the estimated driving force (DF_*Cl*_) for individual neurons. Blue symbols represent cells with a negative DF_*Cl*_, red symbols cells with a positive DF_*Cl*_. Note the obvious correlation between both values. **(D)** Average [Cl^-^]_i_ changes observed at different intervals after GDP termination for all cells with a negative DF_*Cl*_. In these neurons a significant [Cl^-^]_i_ decrease occurs that peaked 2 s after GDP termination. **(E)** Average [Cl^-^]_i_ changes observed at different intervals after GDP termination for all cells with a positive DF_*Cl*_. In these neurons a significant [Cl^-^]_i_ increase occurs. **(F)** Plot of [Cl^-^]_i_ against the frequency of GDPs. No significant correlation between both values was detected. Data points in **(D,E)** represent mean ± SEM, numbers of experiments are given in the diagrams, ^∗^ indicates a *p*-level of <0.05 and ^∗∗^ indicates a *p*-level of <0.01.

These experiments demonstrated that obvious changes in the [Cl^-^]_i_ were observed within the first 1–5 s after a GDP (Figure 4A). On the other hand, the average [Cl^-^]_i_ decreased 1–2 s after a GDP only by 6.1 ± 3.0 mM (*n* = 16 cells, *p* = 0.06). However, in individual neurons considerable [Cl^-^]_i_ changes occurred during this interval (Figure 4B). From this figure it is obvious that GDPs led to a [Cl^-^]_i_ decrease in neurons that had initially a high [Cl^-^]_i_, while they increased [Cl^-^]_i_ in neurons with a low [Cl^-^]_i_.

In order to quantify these different responses, we next estimated the driving-force of Cl^-^ (DF_Cl_) during a GDP from the difference between the average E_m_ during a GDP and E_Cl_. Regression analysis for the [Cl^-^]_i_ changes against the DF_Cl_ revealed a significant (*R*^2^ = 0.713, *F* = 49.74, *p* < 0.001) correlation between DF_Cl_ and the [Cl^-^]_i_ changes after a GDP (Figure 4C). Therefore we subdivided neurons in groups with positive DF_Cl_ and negative DF_Cl_, respectively. In both groups a significant [Cl^-^]_i_ change occurred after a GDP (Figures 4D,E). In the neurons with positive DF_Cl_ the maximal [Cl^-^]_i_ decrease in the first 2 s after a GDP amounted to 12.4 ± 3.4 mM (*n* = 10, *p* = 0.0057, Figure 4D), while GDPs induced in the neurons with negative DF_Cl_ a relatively long lasting [Cl^-^]_i_ increase (Figure 4E), which 2 s after a GDP amounted to 4.4 ± 0.9 mM (*n* = 6, *p* = 0.0039). Further analysis suggested that neither the initial [Cl^-^]_i_, nor the distribution of neurons showing Cl^-^ influx/efflux or the amount of GPD-induced [Cl^-^]_i_ changes depend on the age of the animals (data not shown).

### Inhibition of GDP Activity Reduces [Cl^-^]_i_

Since these results demonstrated that GDPs lead to long lasting [Cl^-^]_i_ changes, which in particular in case of [Cl^-^]_i_ increases showed no evident back regulation in the first 10 s, we postulated that ongoing GDP activity can contribute to the initial [Cl^-^]_i_ of immature hippocampal CA3 pyramidal neurons. Indeed, after inhibition of GDP activity for ≥30 min by bath application of 10 μM CNQX (Bolea et al., 1999), the average [Cl^-^]_i_ amounted to 24.7 ± 2.9 mM (*n* = 8), which is significantly (*p* = 0.0035) smaller than the initial [Cl^-^]_i_ under control conditions (38.1 ± 3.2 mM, *n* = 36). In addition, the CV in the [Cl^-^]_i_ droped from 0.51 under control conditions to 0.33 in the presence of CNQX, indicating a more homogenous distribution of [Cl^-^]_i_. In summary, these results indicate that ongoing GDP activity led in general to an increased [Cl^-^]_i_, suggesting that the association of a high Cl^-^ conductance and a depolarized E_m_ during a GDP in combination with slow passive efflux rates of Cl^-^ favors an accumulation of Cl^-^ in these neurons. In order to evaluate whether the frequency of GDP activity influences [Cl^-^]_i_ of CA3 pyramidal neurons under control conditions, the average occurrence of GDPs was plotted against the initial [Cl^-^]_i_ in these cells (Figure 4F). This analysis revealed that initial [Cl^-^]_i_ and the occurrence of GDPs are not correlated (*R*^2^ = 0.036, *F* = 0.72, *p* = 0.407). This observation indicates that the [Cl^-^]_i_ of an individual cell cannot directly be related to the frequency of GDP activity in the slices. In addition, neither the amplitude of GDPs not their duration are significantly correlated with [Cl^-^]_i_ (data not shown).

### Compartmental Modeling

**FIGURE 5:**
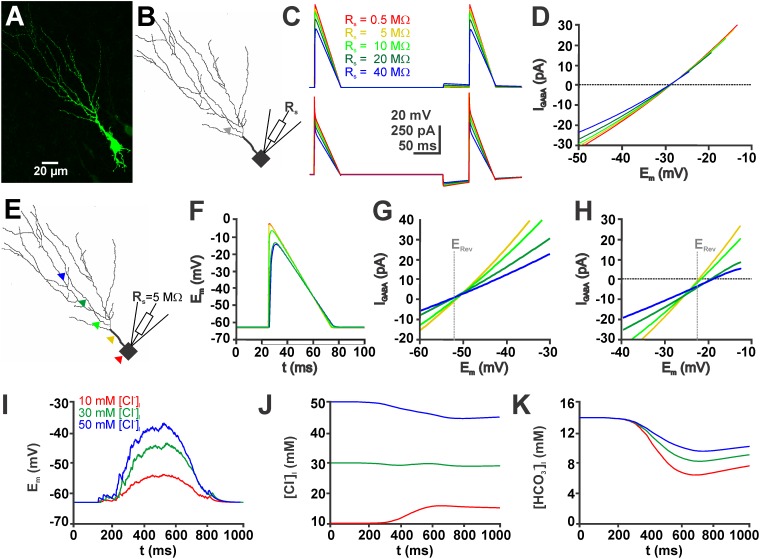
Simulations in a biophysically realistic compartmental model of a CA3 pyramidal cell replicate the dependence of GDP-induced [Cl^-^]_i_ changes on initial [Cl^-^]_i_. **(A)** Biocytin labeled CA3 neuron in a P4 mouse slice. **(B)** Somatodendritic reconstruction of this cell. The gray arrowhead indicates the site of the GABAergic synapse used in **(C,D)**. Voltage-clamp was simulated using the single-electrode voltage-clamp process of NEURON. **(C)** Voltage (top) and current (bottom) traces of voltage ramps applied in the absence and presence of GABA using five different values for the access resistance (R_s_). **(D)** Current-voltage plot of the GABAergic currents (I_GABA_, as archived by subtraction of the current traces) at different R_s_ values. Note that although the slope of the curves clearly depends on Rs, the currents reverse at comparable values. **(E)** Location of five different GABAergic synapses (color coded) on the reconstructed neurons. **(F)** Voltage responses during a voltage ramp at the sites of GABA application (as indicated in **E**). Note the apparent dendritic filtering. **(G)** Current-voltage plot upon GABAergic stimulation at the sites illustrated in **(E)** for a neuron with 10 mM [Cl^-^]_i_. **(H)** Current-voltage plot upon GABAergic stimulation for a neuron with 50 mM [Cl^-^]_i_. The gray line in **(G,H)** marks the calculated reversal potential (E_Rev_). Note the apparent divergence of the measured E_Rev_ at distal application sites. **(I)** Voltage trace of a simulated GDP (see section “Materials and Methods” for details) using three different initial [Cl^-^]_i_. **(J)** GDP-induced [Cl^-^]_i_ changes at different initial [Cl^-^]_i_. Note the Cl^-^ influx at low [Cl^-^]_i_, Cl^-^-efflux at high [Cl^-^]_i_ and small [Cl^-^]_i_ changes at intermediate [Cl^-^]_i_. **(K)** GDP-induced [HCO_3_^-^]_i_ changes under conditions with different initial [Cl^-^]_i_. Note that HCO_3_^-^ influx depend on [Cl^-^]_i_.

The observation that R_in_ was higher and the membrane capacitance was lower in the gramicidin-perforated patch-clamp recordings suggests that the access resistances (R_s_) was under perforated-patch conditions higher than in whole-cell recordings. To investigate to which extend such differences in R_s_ can affect the determination of [Cl^-^]_i_ we employed compartmental modeling using a reconstructed immature CA3 pyramidal neuron (Figures 5A,B, see section “Materials and Methods” for details)_._ Therefore we estimated the influence of R_s_ on the determination of E_GABA_ using a voltage-ramp protocol. Implementation of a voltage ramp protocol via a simulated single-electrode process with defined R_s_ values of 0.5, 5, 10, 20, and 40 MΩ on a neuron with a [Cl^-^]_i_ of 30 mM led to E_GABA_ values of -28.6, -28.7, -28.8, -28.9, and -29.0 mV, respectively (Figures 5B–D). This simulation suggests that E_GABA_ is only marginally affected by R_s_.

Next we used the compartmental modeling to estimate to which extend space-clamp problems will affect the determination of [Cl^-^]_i_ by E_GABA_. These simulations revealed that the E_m_ values during the voltage ramp are attenuated in distant dendrites (Figures 5E,F). Accordingly, E_GABA,_ determined by a voltage ramp applied via a simulated single-electrode (Rs = 5 MΩ) was shifted in the distal dendrites (Figures 5G,H). In a simulated neuron with a defined and static [Cl^-^]_i_ of 50 mM the determined [Cl^-^]_i_ were shifted to higher values. If the GABA synapse was located in the most proximal dendrite a [Cl^-^]_i_ of 50.8 mM was determined, while if the GABA synapse was located in a distal dendrite the estimated [Cl^-^]_i_ increased to 59.1 mM. A similar shift was observed for neurons with a defined [Cl^-^]_i_ of 10 mM. Here, stimulation at proximal synapse resulted in a determined [Cl^-^]_i_ of 10.4 mM and at distal dendrites to a determined [Cl^-^]_i_ of 9.6 mM. These experiments indicate that space-clamp problems can considerably affect [Cl^-^]_i_ calculated from E_GABA_.

Finally, we used compartmental modeling to estimated the GDP-induced [Cl^-^]_i_ changes (Figures 5I,J). These *in silico* experiments revealed that simulated GDP activity induced a [Cl^-^]_i_ decrease by 5.5 mM in neurons with an initial high [Cl^-^]_i_ of 50 mM, a [Cl^-^]_i_ decrease by 1.1 mM in neurons with an intermediate [Cl^-^]_i_ of 30 mM, and a [Cl^-^]_i_ increase by 5.7 mM in neurons with an initial low [Cl^-^]_i_ of 10 mM. In addition, this model also allowed to estimate the [HCO_3_^-^]_i_ changes that occur by activation of GABA_A_ receptors during a GDP (Figure 5K). These modeling studies indicate that the [Cl^-^]_i_ changes were paralleled by a considerable [HCO_3_^-^]_i_ decrease to 6.5 mM in the high [Cl^-^]_i_ neurons, to 8.3 mM in the intermediate [Cl^-^]_i_ neurons, and to 9.6 mM in the high [Cl^-^]_i_ neurons (Figure 5K). In summary, these results suggest that in addition to [Cl^-^]_i_, also [HCO_3_^-^]_i_ shows a considerable ionic plasticity, leading to a decreased DF_HCO3_.

## Discussion

The main aim of the present study was to reveal, whether GDPs lead to detectable [Cl^-^]_i_ changes. The main findings of this study can be summarized as follows: (i) The [Cl^-^]_i_ of CA3 pyramidal neurons in immature hippocampal slices displays a considerable heterogeneity, ranging from 13 to 70 mM. (ii) GDPs induced considerable [Cl^-^]_i_ shifts by several mM, with either Cl^-^-effluxes or Cl^-^-influxes occurring in individual cells. (iii) The direction and amount of the GDP-mediated [Cl^-^]_i_ shifts depends on the DF_Cl_ during the GDP. (iv) Inhibition of GDP activity results in a decrease of the steady-state [Cl^-^]_i_. We conclude from these results that the Cl^-^-fluxes associated with GABAergic activity during GDPs induced significant [Cl^-^]_i_ changes, which attenuate excitatory GABAergic responses and influence the initial [Cl^-^]_i_ in hippocampal neurons.

The [Cl^-^]_i_ in CA3 neurons recorded by perforated patch recordings revealed an obvious large scatter in the present study. From these values it could be assumed that both, inhibitory and excitatory GABAergic responses coexist in CA3 neurons at the same developmental stage, as has been reported in other brain structures (Chavas and Marty, 2003). However, this large scatter of [Cl^-^]_i_ is in line with a series of previous publications that reported similar distributions in the initial [Cl^-^]_i_ of immature hippocampal (Dzhala et al., 2012), but also neocortical neurons (Glykys et al., 2009; Sato et al., 2017). On the other hand, Valeeva et al. (2013) described considerably smaller [Cl^-^]_i_ using non-invasive electrophysiological measurements. In accordance with the established role of NKCC1 for the maintenance of a high [Cl^-^]_i_ in neurons (Rohrbough and Spitzer, 1996; Blaesse et al., 2009), bath application of the loop diuretic bumetanide significantly decreased [Cl^-^]_i_ and also reduced the scatter in [Cl^-^]_i_.

We could not exclude, that part of the rather large scatter in the initial [Cl^-^]_i_ observed in the present publication may to some extent be related to perforated-patch recordings from neurons that have been affected by the slicing procedure (Dzhala et al., 2012). In addition, our modeling studies demonstrate that [Cl^-^]_i_ determined from E_GABA_ are critically affected by space-clamp problems within the dendritic compartment. Thus we cannot exclude that a considerably part of the scatter in [Cl^-^]_i_ and in particular the high [Cl^-^]_i_ in some neurons are caused by insufficient voltage-clamp conditions at the site of GABAergic stimulation. However, while this obstruction influences the absolute [Cl^-^]_i_ values, it doesn’t affect the main observation of this study, that GDPs induce considerable [Cl^-^]_i_ shifts. Because we used identical stimulation parameters before and after a GDP, the influence of space-clamp errors should be identical for both [Cl^-^]_i_ values. In contrast, R_s_ seems to have only a marginal effect on the determination of E_GABA_, which is mainly caused by the fact that during a voltage ramp protocol I_GABA_, and thus the voltage-error caused by R_s_, is minimal at E_GABA_.

And finally, our modeling studies demonstrate that the [Cl^-^]_i_ changes were paralleled by a considerable [HCO_3_^-^]_i_ decline of several mM. These putative [HCO_3_^-^]_i_ changes were not considered in the calculation of [Cl^-^]_i_ from E_GABA_. If these estimated reduced [HCO_3_^-^]_i_ was used in the Goldman Equation we calculated slightly reduced [Cl^-^] values of 48.1 mM for a [Cl^-^]_i_ of 50 mM, of 27.5 mM for a [Cl^-^]_i_ of 30 mM and of 6.7 mM for a [Cl^-^]_i_ of 10 mM. However, the validity of such corrections for [HCO_3_^-^]_i_ shifts critically depend on the [HCO_3_^-^]_i_ values in individual neurons, which cannot be determine experimentally. Because our modeling results indicate that consideration of these [HCO_3_^-^]_i_ changes for the calculation of [Cl^-^]_i_ resulted in a slight, but consistent underestimation of [Cl^-^]_i_, we suggest that the main implications of the present results, namely that GDPs lead to considerable [Cl^-^]_i_ changes and that the direction of these [Cl^-^]_i_ changes depends on the initial [Cl^-^]_i_, are still valid. On the other hand, the absolute values for these changes represents probably only an estimate of the [Cl^-^]_i_ changes in the dendritic compartment.

In any way, the mean [Cl^-^]_i_ value of our recordings is in good agreement with the values expected to be recorded in neurons residing sufficiently deep below the slice surface to be unaffected by the slicing procedure (Dzhala et al., 2012) and the in-toto, unsliced hippocampal preparation shows a comparable wide distribution of [Cl^-^]_i_ between 5 and 50 mM (Dzhala et al., 2012). In addition, we considered the first [Cl^-^]_i_ value that was determined in a cell as initial [Cl^-^]_i_. However, we cannot exclude that this cell experienced a GDP before the recording interval started (about 30–60 s before [Cl^-^]_i_ determination). Finally, our results revealed that inhibition of GDP activity reduced not only the initial [Cl^-^]_i_, but also the variance in these values, indicating that probably also different frequency or different amounts of GABAergic inputs during GDPs may contribute to the scatter in [Cl^-^]_i_.

From the observation that E_GABA_ and [Cl^-^]_i_ was increased in superficial neurons of the traumatized slice surface, it has been suggested that part of the excitatory GABAergic action may represent a “slicing artifact” (Dzhala et al., 2012). However, GDPs have also been observed in whole-hippocampal preparations (Leinekugel et al., 1998), which excludes that they represent “slicing artifacts” caused by artificially increased [Cl^-^]_i_ in traumatized neurons (Valeeva et al., 2013). In fact, GDPs are also present under *in vivo* conditions (Khazipov et al., 2001; Leinekugel et al., 2002), suggesting that GABA_A_ receptors may provide the excitatory drive required to trigger GDPs also *in vivo*. These observations in the immature hippocampus are in contrast to recent *in vivo* recordings from immature neocortical neurons, which revealed that GABA_A_ receptor-mediated responses are indeed depolarizing, but most probably provide an inhibitory effect on immature neocortical neurons (Kirmse et al., 2015; Valeeva et al., 2016).

While the GABAergic conductance underlying GDPs determined in our study is in agreement with published values (Khalilov et al., 2015), the glutamatergic component is considerably higher than the published values. In addition, there is an obvious discrepancy between our conclusion that comparable numbers of GABAergic and glutamatergic synaptic inputs underlie a GDP (drawn from the comparison of charge transfer), while the detection of synaptic inputs from the first derivative of the current trace at -30 mV revealed much less identified glutamatergic synaptic events. However, the latter method detects clearly not all events, but, in particular during the phase of maximal inputs, only the most prominent ones. Therefore we assume that a considerable portion of GABAergic events and a larger fraction of the glutamatergic events remained undetected in this analysis. Nevertheless, we still consider that this analysis provides a good estimate for the temporal relation between GABAergic and glutamatergic inputs. Our findings are also in good agreement with previous studies demonstrating that GABAergic inputs precede the onset of glutamatergic inputs (Mohajerani and Cherubini, 2005; Khalilov et al., 2015).

The main observation of the present study is that GDPs induced considerable [Cl^-^]_i_ shifts. Such ionic plasticity in [Cl^-^]_i_ transients has been found after a variety of pathophysiological impacts (Lillis et al., 2012; Kaila et al., 2014b; Sato et al., 2017) and physiological processes (Bracci et al., 2001; Chub et al., 2006; Gonzalez-Islas et al., 2010; Kolbaev et al., 2011b). While in the present study the overall effect was partially masked by the variability in initial [Cl^-^]_i_, considerable GDP-induced [Cl^-^]_i_ shifts were observed in individual neurons. Due to the large variability in the initial [Cl^-^]_i_ and thus in the DF_Cl_ both, Cl^-^-influx and Cl^-^-efflux were mediated by activated GABA_A_ receptors. Therefore, GDPs induced a [Cl^-^]_i_ reduction in neurons with a negative DF_Cl_ (resulting mainly from a high [Cl^-^]_i_) and an [Cl^-^]_i_ increase in neurons with a positive DF_Cl_ (resulting mainly from a low [Cl^-^]_i_). The [Cl^-^]_i_ increase of ∼4 mM observed in low [Cl^-^]_i_ neurons after a GDP in the present study is in line with the ∼ 4 mM [Cl^-^]_i_ increase induced in the dendritic compartments of adult hippocampal neurons upon moderate electrical stimulation (43 single pulses at 23 Hz) (Berglund et al., 2008).

In order to evaluate whether the recorded GABAergic currents during a GDP can indeed lead to the observed GDP-induced [Cl^-^]_i_ changes, we implemented a biophysically realistic compartmental model that allows dynamic [Cl^-^]_i_ and [HCO_3_^-^]_i_ changes. Simulating GDPs in a reconstructed immature CA3 pyramidal cell with a burst of GABAergic inputs that resembles the unitary GABAergic conductance and the total charge transfer during a GDP resulted in considerable dendritic [Cl^-^]_i_ changes. The amount of these [Cl^-^]_i_ changes is roughly comparable to the experimentally determined values. Our modeling studies also demonstrate that the [Cl^-^]_i_ changes were paralleled by considerably [HCO_3_^-^]_i_ decreases by several mM. Such a [HCO_3_^-^]_i_ decrease will lead to a decreased DF_HCO3_ and thus affects E_GABA_. This observation supports the concept that also [HCO3^-^]_i_ changes contribute to ionic plasticity after GABAergic stimulation (Staley and Proctor, 1999; Blaesse et al., 2009; Raimondo et al., 2012), in particular in the immature brain where the replenishment of [HCO_3_^-^]_i_ via carbonic anhydrase VII is reduced (Rivera et al., 2005).

The GDP-induced shifts in [Cl^-^]_i_ affect the DF_Cl_ and thus the functional consequences of subsequent GABA_A_ receptor activation. However, it is important to consider that the [Cl^-^]_i_ of the arbitrary neuron investigated by patch-clamp is most probably not decisive for the generation of GDP activity in a slice, but that GDPs are initiated in few hub neurons (Bonifazi et al., 2009; Wester and McBain, 2016). While the [Cl^-^]_i_ increase in low [Cl^-^]_i_ neurons attenuates the inhibitory effect of GABA in these neurons (Staley and Proctor, 1999; Raimondo et al., 2012), the [Cl^-^]_i_ decrease in high [Cl^-^]_i_ neurons reduced depolarizing GABAergic responses and their putative excitatory effect (Kolbaev et al., 2011a,b). Although the small GDP-induced [Cl^-^]_i_ decline in the whole population of recorded neurons suggests that these [Cl^-^]_i_ shifts may only marginally reduce the global excitatory effect of GABA on network excitability, the [Cl^-^]_i_ decline in the high [Cl^-^]_i_ neurons will clearly attenuate the excitatory potential of GABA in these cells. In addition, the GDP-induced [HCO_3_^-^]_i_ decline, which was suggested from our modeling studies, reduces the inwardly directed DF_HCO3_ and would thus additionally attenuate an excitatory effect of GABA.

As the initiation of GDPs (Sipila et al., 2005) as well as the positive feedback during the “onset” phase (Khalilov et al., 2015) depend on excitatory GABAergic inputs, we propose that these high [Cl^-^]_i_ neurons may be particularly relevant for the establishment of GDPs. Therefore we assume that the transient, GDP-mediated [Cl^-^]_i_ decline in CA3 pyramidal neurons with high [Cl^-^]_i_ will considerably attenuate the excitatory effect of GABA during a GDP. This effect adds to the dynamic model of Khalilov et al. (2015), which demonstrated that during the peak (or “catharsis”) phase of the GDP DF_Cl_ becomes positive, thus providing an inhibitory effect that is essential to limit excitatory influences.

Interestingly, it has been observed that interneurons of the mature hippocampus are much more susceptible to activity-dependent shifts in E_GABA_ than pyramidal neurons (Lamsa and Taira, 2003), suggesting that ionic plasticity during a GDP is more pronounced in these cells. Therefore the typically shunting depolarization provoked by GABA_A_ receptors in hippocampal interneurons (Banke and McBain, 2006), might be shifted to excitatory effects during a GDP. And since a subpopulation of GABAergic interneurons serve as hub neurons that orchestrate GDP activity (Bonifazi et al., 2009; Picardo et al., 2011), the resulting decline in the GABAergic inhibitory drive on GABAergic interneurons will probably impact the frequency of GABAergic inputs to pyramidal neurons during GDPs.

In addition, the [Cl^-^]_i_ dynamics and the resulting decreased excitatory drive of GABAergic inputs may contribute to the termination phase of the GDP, in addition to other processes that have been shown to terminate GDPs (Sipila et al., 2006a; Khalilov et al., 2017). The calculation from Khalilov et al. (2015) proposed depolarizing GABAergic current during the repolarization phase, because their assumption of a constant [Cl^-^]_i_ resulted in stable E_GABA_ values. Therefore DF_GABA_ will become negative when E_m_ falls below E_GABA_ during the repolarization. The collapse of [Cl^-^]_i_ during a GDP observed in the present study may explain a reduced GABAergic depolarization/excitation during this phase, which will support the termination of a GDP.

In the developing spinal cord GABA-dependent spontaneous neuronal activity induces a collapse of the Cl^-^ gradient, which temporarily attenuates the excitatory drive provided by GABA_A_ receptors, and the subsequent slow Cl^-^ accumulation that reestablishes GABAergic excitation underlies the low frequency of this recurrent activity (Chub and O’Donovan, 2001; Chub et al., 2006). Thus the dynamics of hippocampal [Cl^-^]_i_ homeostasis after a GDP may also determine the frequency of GDPs. The breakdown of [Cl^-^]_i_ in the high [Cl^-^]_i_ neurons and in hub neurons will reduce excitatory GABAergic responses (Kolbaev et al., 2011a), which not only contributes to the shutoff of GDPs, but also prevents the generation of new excitatory network events. With the [Cl^-^]_i_ re-accumulation via NKCC1, GABAergic responses regain their excitatory potential. At the time point when the net GABAergic effect becomes sufficiently excitatory again, it can contribute to the generation of the next GDP. We assume that neurons with a low [Cl^-^]_i_ do not contribute to the initiation of GDP activity, thus the GDP-induced [Cl^-^]_i_-increase in these neurons probably doesn’t play an essential role in the generation of GDPs.

The observation that the peak of [Cl^-^]_i_ changes occurred with several seconds delay to the end of the GDP most probably reflects the time required for a diffusional equilibration within the dendrites (Kuner and Augustine, 2000) and is in line with previously reported delays of 2–5 s between electrical stimulation and the peak of dendritic [Cl^-^]_i_ responses (Berglund et al., 2008, see also Jedlicka et al., 2011). While this rather slow propagation of [Cl^-^]_i_ transients limits the spatial extend of ionic plasticity, it doesn’t exclude that GDPs induces in the vicinity of GABAergic synapses sufficiently high [Cl^-^]_i_ alterations to affect GABAergic transmission.

The KCC2-dependent back-regulation of [Cl^-^]_i_ after an activity dependent increase in mature neurons occurred within several seconds (Lamsa and Taira, 2003; Jin et al., 2005). However, as the Cl^-^-loader NKCC1 probably modulates a less effective transmembrane transport, [Cl^-^]_i_ back regulation upon activity-dependent Cl^-^ loss in immature neurons seems to take longer time, even up to some minutes (Achilles et al., 2007). Thus the observed slow recovery of the GDP-induced Cl^-^-loss in high [Cl^-^]_i_ neurons is in agreement with the relatively slow time constant of NKCC1 mediated Cl^-^ uptake. The recovery upon GDP-induced [Cl^-^]_i_ increases in the low [Cl^-^]_i_ group appeared to be even slower, indicating that it is mainly mediated by [Cl^-^]_i_ diffusion toward the soma and the ineffective passive transmembrane fluxes observed in immature hippocampal neurons. This observation was supported by the slow [Cl^-^]_i_ decline observed in the presence of the NKCC1 inhibitor bumetanide.

The fact that recovery rates after [Cl^-^]_i_ increases were slower than after [Cl^-^]_i_ losses also suggests that ongoing GDP activity may increase basal [Cl^-^]_i_. Indeed, we observed that inhibition of GDP activity significantly reduces initial [Cl^-^]_i_. This result demonstrates that the [Cl^-^]_i_ in immature neurons depends substantially on GABAergic activity due to the slow kinetics of activity dependent [Cl^-^]_i_ transients. However, GDPs may also effect [Cl^-^]_i_ homeostasis by post-translational modification of KCC2 and NKCC1 transporters (Blaesse et al., 2009; Kaila et al., 2014a).

In summary, our results indicate that the [Cl^-^]_i_ and thus the physiological effects of GABAergic inputs critically depend on prior GABAergic activity. Therefore, in immature hippocampal neurons ionic plasticity must be considered to explain [Cl^-^]_i_ and thus the functional state of GABAergic transmission at each single time point. These observations also foster the concept that neuronal [Cl^-^]_i_ has to be considered as a state- and compartment-dependent parameter of individual cells (Wright et al., 2011).

## Author Contributions

WK and HL designed this study. AL performed all recordings. AL and WK analyzed the data. WK, AL, and PJ performed computational modeling. AL, HL, PJ, and WK wrote the manuscript.

## Conflict of Interest Statement

The authors declare that the research was conducted in the absence of any commercial or financial relationships that could be construed as a potential conflict of interest.

## Footnotes

1 www.arduino.cc

2 www.fiji.sc

3 neuron.yale.edu
